# Functional dependence predicts adverse outcomes among geriatric otolaryngology patients better than more complex risk scales: a multivariate analysis of hospitalization risks on elderly group

**DOI:** 10.3389/fmed.2025.1690442

**Published:** 2025-12-11

**Authors:** Małgorzata Wierzbicka, Dorota Świątek, Andrzej Porębski, Jarosław Markowski, Katarzyna Ciuba, Maria Makuszewska, Kazimierz Niemczyk, Karolina Dżaman, Katarzyna Czerwaty, Bogusław Mikaszewski, Dominik Stodulski, Maciej Kawczyński, Magdalena Rękawek, Małgorzata Wierzchowska, Joanna Czech, Kamila Sroka, Wioletta Pietruszewska

**Affiliations:** 1 Department of Otolaryngology, Regional Specialist Hospital Wrocław, Research and Development Centre, Wrocław, Poland; 2 Faculty of Medicine, Wrocław University of Science and Technology, Wrocław, Poland; 3 Institute of Human Genetics, Polish Academy of Sciences, Poznań, Poland; 4 Faculty of Law and Administration, Jagiellonian University, Kraków, Poland; 5 Department of Laryngology, Faculty of Medical Sciences in Katowice, Medical University of Silesia in Katowice, Katowice, Poland; 6 Department of Otorhinolaryngology Head and Neck Surgery, Medical University of Warsaw, Warsaw, Poland; 7 Department of Otolaryngology, Centre of Postgraduate Medical Education, Warsaw, Poland; 8 Department of Otolaryngology, Faculty of Medicine, Medical University of Gdańsk, Gdańsk, Poland; 9 Department of Otolaryngology, Pomeranian Medical University, Szczecin, Poland; 10 Department of Otolaryngology, Phoniatrics and Audiology, Nicolaus Copernicus University in Torun, Collegium Medicum in Bydgoszcz, Bydgoszcz, Poland; 11 Department of Otolaryngology, Laryngological Oncology and Maxillofacial Surgery, University Hospital No2 in Bydgoszcz, Bydgoszcz, Poland; 12 Department of Otolaryngology Head Neck Oncology, Medical University of Łódź, Łódź, Poland

**Keywords:** geriatrics, otorhinolaryngology, geriatric assessment, functional assessment, aging, comorbidities, prolonged hospital length of stay

## Abstract

**Introduction:**

As the population of adults aged 80 years and older continues to grow, otorhinolaryngology departments face increasing demands to manage complex and vulnerable older patients. Identifying reliable predictors of adverse outcomes in this population is essential for optimizing care.

**Methods:**

In this multicenter retrospective study, data from 426 hospitalized patients aged ≥ 80 years were collected across eight university hospitals. The study investigated three clinical outcomes: prolonged hospitalization, 30-days serious complications, and 90-days functional decline. Explanatory variables included, inter alia, functional status measures and traditional risk assessment tools such as the ASA score, Caprini score, and Charlson Comorbidity Index (CCI). Potential predictors of adverse outcomes were examined using univariate tests, as well as multivariate logistic regression modeling.

**Results:**

Physical inactivity (*p* < 0.012), dependence in daily functioning (*p* < 0.009), and impaired food intake (*p* < 0.003) consistently predicted prolonged hospitalization, serious complications, and functional decline. The three variables describing functional status proved to be the most significant predictors of adverse outcomes among the variables included in the study. Most of the traditional assessment tools—including the ASA and Caprini scales—showed limited predictive value compared to the functional status variables, whereas CCI provided additional prognostic information.

**Discussion:**

Functional status indicators, particularly mobility, dependence in activities of daily living, and food intake, emerged as strong and consistent predictors of major adverse outcomes in geriatric otorhinolaryngology inpatients. These findings support the integration of functional measures into routine risk stratification to better identify high-risk older adults and guide more individualized clinical management strategies.

## Introduction

Geriatric otorhinolaryngology has emerged as a specialized field in response to the growing healthcare challenges posed by increasing life expectancy, particularly in developed countries (1–3). The World Health Organization (WHO) defines elderly individuals as those aged 65 years or older, while the National Institute on Aging provides a more nuanced classification, subdivides this group into “young old” (65–74 years), “older old” (75–85 years), and “oldest old” (85+) (4, 5).^1^ The rapid demographic shifts have significantly increased the demand on healthcare systems, making the provision of adequate care for elderly patients a pressing concern (1). Despite the growing relevance of this field (6), the reasons for hospitalization in geriatric otorhinolaryngology remain underexplored.

Simultaneously, and yet in a complementary contrast, elderly patients are particularly vulnerable to prolonged hospital stays (PH), often necessitating additional resources such as consultations and advanced nursing interventions (7, 8). This trend contributes to rising healthcare costs, with extended hospitalizations being a major factor (9, 10). Consequently, reducing hospital stays has become a key strategy to ensure sustainable healthcare delivery without compromising quality (8, 11). Given the anticipated substantial rise in number of hospitalized seniors, it is crucial to thoroughly understand their causes, evaluate the general health status of incoming patients, and identify factors contributing to extended hospitalizations.

In otorhinolaryngology head and neck surgery, patients aged 80 and above face unique risks that influence both their length of stay and discharge processes. Medical comorbidities which are more prevalent in this age group, significantly impact treatment outcomes and recovery (12, 13). Multimorbidity is frequently accompanied by polypharmacy, which further complicates clinical management. To address these complexities, tools such as the Charlson Comorbidity Index (CCI), Caprini score, American Society of Anesthesiologists Physical Status Classification System (ASA), and risk assessment for venous thromboembolism (VTE) have been developed to quantify comorbidities (14) and predict hospitalization outcomes more effectively (12, 15). However, there is still a need for geriatric-specific models that combine functional status with comorbidity scores to improve prediction of adverse outcomes. Traditional tools alone may not fully capture the clinical complexity of very old otolaryngology patients.

Thus, this multicenter study aims to characterize the population of hospitalized patients aged 80 years and above in eight university otorhinolaryngology departments, including the distribution of primary diagnoses according to ICD-10 classification. The objective of this study is to elucidate the associations between clinical, demographic, and functional characteristics and three specific adverse outcomes: PH, serious complications within 30 days, and functional decline at 90 days. Furthermore, the analysis seeks to delineate intervariable relationships to identify which factors exhibit the strongest associations with these adverse events. A further objective is to assess the predictive utility of established risk assessment tools—such as CCI, ASA and Caprini Score—in identifying patients at increased risk of adverse outcomes in this elderly otolaryngology cohort.

## Materials and methods

### Sample characteristics

The study was approved by the Bioethics Committee prior to data collection. This retrospective observational study included data collected over a 12-month period, from September 2023 to August 2024, across eight university-affiliated otorhinolaryngology departments in Poland. Participating centers were located in Wrocław (two hospitals), Gdańsk, Bydgoszcz, Szczecin, Warsaw, Katowice, and Łódź. All patients aged 80 years or older who were hospitalized in otorhinolaryngology wards during the study period were eligible for inclusion. In cases of multiple hospital admissions for the same patient, only the primary hospitalization—defined as the one with the greatest relevance to the overall course of treatment—was included in the analysis.

The sample consists of 426 individuals aged 80–97 years with an equal number of males and females. The mean age of the patients was 84.4 years (SD = 3.584, 1st quartile = 82, median = 84, 3rd quartile = 87), with the distribution of this variable being slightly positively skewed (skewness coefficient: 0.91). The sample predominantly consists of individuals aged 80–83 years (half of the patients fall within this age range), with a small number of participants in very advanced ages deviating significantly from the mean (10% of patients in the sample had 90 years or more, 3%—93 years or more).

The majority of patients are independent, while approximately one in eight requires assistance with feeding, either through a caregiver or via a gastric tube or gastrostomy. Two-thirds of patients take more than three medications. Only 6% of individuals declared consuming alcohol, yet only 60% of patients in the sample have never smoked in their lifetime. 8% of participants are active smokers (47 observations were missing). Approximately one-fourth of patients was not physically active. For detailed characteristics of the sample, see Supplementary Table S1.

### Definition of variables and methodological assumptions

As the primary outcome measure, best reflecting the presence of risk factors in elderly patients, the occurrence of PH, 30-days serious complications, and 90-days functional decline were selected. PH were defined based on economic criteria as hospitalizations exceeding the median hospital stay by more than one and a half interquartile ranges (IQR: difference between the third and first quartiles). With a median of 3 days and IQR of 4, hospital stays lasting ≥ 10 days (that is, higher than median + 1.5 IQR) were classified as prolonged. This criterion identified 49 cases out of 426 observations (11.5%). Moreover, 16 out of 360 cases (4.5%; 66 missing values) were classified as 30-days serious complications and 28 cases out of 277 observations (10.1%; 149 missing values) were classified as 90-days functional decline. 30-days serious complications were measured according to Clavien–Dindo scale, based on review of medical records and discharge summaries. 90-days functional decline was assessed by comparing admission and follow-up documentation on activities of daily living, the level of patient independence from fully independent to fully dependent and subjective assessments of patient’s health made by family members or caregivers. All patients had an outpatient control visit scheduled after hospitalization, during which functional status was reassessed.

Functional status was systematically assessed, as it constitutes a critical determinant of prognosis in this population. Functional status was extracted from clinical records at admission and categorized across three domains. Physical activity was recorded as yes, no, or limited, reflecting the patient’s ability to ambulate independently. “Yes” indicated independent walking without support, “no” indicated immobility or confinement to bed/wheelchair, and “limited” reflected the need for assistance from another person or mobility aids (e.g., crutches). Feeding dependency was assessed through observation, patient or caregiver interview, and medical documentation, using a four-point scale: independent oral intake (normal diet without support), oral intake requiring assistance or specialized food preparations (e.g., nutridrinks), feeding via gastrostomy, and feeding via nasogastric tube. Independence in activities of daily living was determined by interview and classified into three categories: independent—living alone and managing not only basic activities (e.g., bathing, toileting, dressing) but also more complex daily activities (such as shopping, transportation, and paying bills); requiring help—needing assistance several times per week for more complex activities; and fully dependent—requiring daily support with all basic activities. These variables were consistently coded across all eight participating centers to ensure comparability and were subsequently included in univariate and multivariate analyses as potential predictors of adverse outcomes. In addition to three variables operationalizing functional status, the study took into account: gender, polypharmacy, alcohol consumption, smoking, oncological patient status including cancer other than that located in the head and neck area, and oncological family history.

Group comparisons were stratified by PH status, 30-days serious complications and 90-days functional decline. Analytical methods included *χ*^2^ test for categorical or ordinal variables with ≤ 3 categories, Welch’s *t*-test (mean differences, a test without equal variances assumption) and Mann–Whitney-Wilcoxon *U* test (testing P[X > Y] = P[Y > X]; reported only in Supplementary Table S3) for interval or ordinal variables with multiple categories. Effect sizes were reported as Cramer’s *V* for *χ*^2^, Cohen’s *d* for *t*-tests, rank-biserial correlation for Mann–Whitney-Wilcoxon *U* tests.

The statistical modeling approach involved initial construction of three logistic regression models incorporating dependent variable (clinical outcome of interest) and nine explanatory variables: the Caprini score, Charlson Comorbidity Index (CCI), the ASA classification, VTE risk, Vulnerable Elders Survey-13 (VES-13), a frailty scale (mFI-5), and three predictors that proved to be the best in the preceding univariate analysis (i.e., functional status variables). Next, sequential model refinement through backward elimination of non-significant predictors to reduce informational noise from excessive predictors was done, mitigate sample size reduction from missing data, enhance estimation precision. Only final (i.e., reduced) models are reported in the text, i.e., those that contain only significant predictors and have the smallest AIC and the largest pseudo-*R*^2^ possible.

Given the multiple comparison framework, a stringent significance level, *α* = 0.005, was employed, as suggested in recent literature (16, 17). Thus, reported findings primarily highlight associations with *p*-values equal or below 0.005 and include *p*-values near or below 0.01 but higher than 0.005 as “marginally significant.”

## Results

The distribution of primary ICD-10 disease code categories assigned to patients is summarized in Table 1. Neoplasms (codes C and D) were the most frequently assigned categories, accounting for 47% of all codes and 53% of primary codes. Diseases of the ear and mastoid process (H60–H95) and respiratory system diseases (J) were also common, representing 16 and 14% of all codes (as well as primary codes), respectively. Table 2 provides a more detailed breakdown of the most frequently assigned diagnostic codes, highlighting the predominance of malignant neoplasm-related codes, particularly C32 (Malignant neoplasm of the larynx), with a notable emphasis on C32.0 (glottis).

**Table 1:** Frequencies of major ICD-10 categories in the sample, including a division by prolonged hospitalizations.

ICD10 group	Category name	Number of subcategories in the sample	Total		Prolonged hospitalization			
					No		Yes	
			*n*	%	*n*	%	*n*	%
C	Malignant neoplasms	42	150	35.21	123	82.0	27	18.0
D	*In situ* neoplasms; Benign neoplasms; Neoplasms of uncertain or unknown behavior	25	77	18.08	68	88.3	9	11.7
H	Diseases of the eye and adnexa; Diseases of the ear and mastoid process	31	69	16.20	64	92.8	5	7.2
J	Diseases of the respiratory system	26	61	14.32	60	98.4	1	1.6
R	Symptoms, signs and abnormal clinical and laboratory findings, not elsewhere classified	9	25	5.87	23	92.0	2	8.0
K	Diseases of the digestive system	10	20	4.69	17	85.0	3	15.0
Z	Factors influencing health status and contact with health services	2	9	2.11	9	100.0	0	0.0
G	Diseases of the nervous system	4	6	1.41	5	83.3	1	16.7
T	Injury, poisoning and certain other consequences of external causes	3	4	0.94	4	100.0	0	0.0
S	Injury, poisoning and certain other consequences of external causes	2	2	0.47	1	50.0	1	50.0
B	Certain infectious and parasitic diseases	1	1	0.23	1	100.0	0	0.0
L	Diseases of the skin and subcutaneous tissue	1	1	0.23	1	100.0	0	0.0
Q	Congenital malformations, deformations and chromosomal abnormalities	1	1	0.23	1	100.0	0	0.0
Total		157	426	100	377	88.5	49	11.5

**Table 2:** Most frequently occurring ICD-10 codes in the sample (basic and detailed levels) and percent of patients characterized by them.

ICD-10 codes (basic level of disease identification)				ICD-10 codes (detailed level of disease identification)			
Code	Description	*n*	%	Code	Description	*n*	%
C32	Malignant neoplasm of larynx	68	16	C32.0	Malignant neoplasm: Glottis	34	8
C44	Other malignant neoplasms of skin	33	8	C44.2	Malignant neoplasm: Skin of ear and external auricular canal	22	5
D38	Neoplasm of uncertain or unknown behavior of middle ear and respiratory and intrathoracic organs	20	5	H90.3	Sensorineural hearing loss, bilateral	21	5
H90	Conductive and sensorineural hearing loss	22	5	D38.0	Neoplasm of uncertain or unknown behavior: Larynx	17	4
D11	Benign neoplasm of major salivary glands	19	4	D11.0	Benign neoplasm: Parotid gland	19	4
H81	Disorders of vestibular function	18	4	C32.8	Malignant neoplasm: Overlapping lesion of larynx	14	3
J32	Chronic sinusitis	17	4	C32.9	Malignant neoplasm: Larynx, unspecified	13	3
J38	Diseases of vocal cords and larynx, not elsewhere classified	15	4	Z46.8	Fitting and adjustment of other specified devices	13	3
K11	Diseases of salivary glands	14	3	C07	Malignant neoplasm of parotid gland	11	3
Z46	Fitting and adjustment of other devices	14	3	I10	Essential (primary) hypertension	10	2
D14	Benign neoplasm of middle ear and respiratory system	12	3				
H66	Suppurative and unspecified otitis media	12	3				
R04	Hemorrhage from respiratory passages	12	3				
C07	Malignant neoplasm of parotid gland	11	3				
J34	Other disorders of nose and nasal sinuses	11	3				
D48	Neoplasm of uncertain or unknown behavior of other and unspecified sites	10	2				
I10	Essential (primary) hypertension	10	2				
R06	Abnormalities of breathing	10	2				

PH, the primary outcome measure, was analyzed in relation to ICD-10 code categories (Table 1). The prevalence of PH varied across categories, ranging from 0% (Z-group, 0/9 cases) to 18% (C-group, 27/150 cases). However, this relationship can be observed in the sample but its significance cannot be determined due to a large number of groups and very small size of some groups (*χ*^2^(12) = 18.97, *p* = 0.089).

The characteristics of the selected variables—gender, oncological status, and three variables describing patient independence—including a division by PH, 30-days serious complications, and 90-days functional decline and *χ*^2^ test of significance are summarized in Table 3. The statistics and comparisons including all examined variables are provided in Supplementary Table S1. Among the various factors analyzed, nutritional dependency, dependence in daily functioning and physical activity, emerged as particularly influential, showing significant dependency with all three clinical outcomes.

**Table 3:** Descriptive statistics and results of *χ*^2^ tests of independence of the selected variables across groups of clinical outcomes.

Variable	Category	Total	Prolonged hospitalization		30-Days serious complications		90-Days functional decline	
			No	Yes	No	Yes	No	Yes
Gender	Female	213	189	24	168	7	125	10
		50.00%	88.73%	11.27%	96.00%	4.00%	92.59%	7.41%
	Male	213	188	25	176	9	124	18
		50.00%	88.26%	11.74%	95.14%	4.87%	87.32%	12.68%
	Total	426	377	49	344	16	249	28
		100.00%	88.50%	11.50%	95.56%	4.44%	89.89%	10.11%
	*Χ*^2^(df), *p*-value		*Χ*^2^(1) = 0.023, *p* = 0.879		*Χ*^2^(1) = 0.158, *p* = 0.691		*Χ*^2^(1) = 2.114, *p* = 0.146	
	Cramer’s *V*		*V* = 0.007		*V* = 0.021		*V* = 0.087	
Oncological patient	No	219	200	19	174	5	120	10
		52.39%	91.32%	8.68%	97.21%	2.79%	92.31%	7.69%
	Yes	199	169	30	167	10	126	17
		47.61%	84.93%	15.08%	94.35%	5.65%	88.11%	11.89%
	Total	418	369	49	341	15	246	27
		100.00%	88.28%	11.72%	95.79%	4.21%	90.11%	9.89%
	*Χ*^2^(df), *p*-value		*Χ*^2^(1) = 4.126, *p* = 0.042		*Χ*^2^(1) = 1.799, *p* = 0.180		*Χ*^2^(1) = 1.345, *p* = 0.246	
	Cramer’s *V*		*V* = 0.099		*V* = 0.071		*V* = 0.070	
Independence	Independent	307	279	28	265	6	192	13
		72.58%	90.88%	9.12%	97.79%	2.21%	93.66%	6.34%
	Assisted living	91	77	14	65	7	52	8
		21.51%	84.62%	15.39%	90.28%	9.72%	86.67%	13.33%
	Dependent	25	18	7	12	3	3	7
		5.91%	72.00%	28.00%	80.00%	20.00%	30.00%	70.00%
	Total	423	374	49	342	16	247	28
		100.00%	88.42%	11.58%	95.53%	4.47%	89.82%	10.18%
	*Χ*^2^(df), *p*-value		*Χ*^2^(2) = 9.680, *p* = 0.008		*Χ*^2^(2) = 16.356, *p* < 0.001		*Χ*^2^(2) = 43.085, *p* < 0.001	
	Cramer’s *V*		*V* = 0.151		*V* = 0.214		*V* = 0.396	
Food intake	Independently	364	333	31	315	8	232	21
		87.29%	91.48%	8.52%	97.52%	2.48%	91.70%	8.30%
	With other person help	37	29	8	18	7	10	4
		8.87%	78.38%	21.62%	72.00%	28.00%	71.43%	28.57%
	Gastrostomy	9	4	5	8	0	4	3
		2.16%	44.44%	55.56%	100.00%	0.00%	57.14%	42.86%
	Feeding tube	7	3	4	2	1	3	0
		1.68%	42.86%	57.14%	66.67%	33.33%	100.00%	0.00%
	Total	417	369	48	343	16	249	28
		100.00%	88.49%	11.51%	95.54%	4.46%	89.89%	10.11%
	*Χ*^2^(df), *p*-value		*Χ*^2^(3) = 38.369, *p* < 0.001		*Χ*^2^(3) = 41.764, *p* < 0.001		*Χ*^2^(3) = 14.762, *p* = 0.002	
	Cramer’s *V*		*V* = 0.303		*V* = 0.341		*V* = 0.231	
Physical activity	Yes	147	141	6	131	1	92	4
		37.79%	95.92%	4.08%	99.24%	0.76%	95.83%	4.17%
	Limited	138	113	25	104	7	82	9
		35.48%	81.88%	18.12%	93.69%	6.31%	90.11%	9.89%
	No	104	86	18	78	8	61	15
		26.74%	82.69%	17.31%	90.70%	9.30%	80.26%	19.74%
	Total	389	340	49	313	16	235	28
		100.00%	87.40%	12.60%	95.14%	4.86%	89.35%	10.65%
	*Χ*^2^(df), *p*-value		*Χ*^2^(2) = 15.596, *p* < 0.001		*Χ*^2^(2) = 8.972, *p* = 0.011		*Χ*^2^(2) = 10.894, *p* = 0.004	
	Cramer’s *V*		*V* = 0.200		*V* = 0.165		*V* = 0.204	

Specifically, 28% of dependent in daily functioning patients experienced PH, 20% suffered serious complications within 30 days, and 70% showed functional decline at 90 days. In contrast, these adverse outcomes were less common among patients living with assistance (15, 10, and 13%, respectively) and rarest among independent patients (9, 2, and 6%, respectively). The differences in PH rates were marginally significant, while other differences were strongly statistically significant (*V* = 0.151, *p* = 0.008; *V* = 0.214, *p* < 0.001; *V* = 0.396, *p* < 0.001).

Similarly, patients who were able to eat independently had markedly better outcomes: only 9% had PH, 2% had 30-days complications, and 8% experienced 90-days functional decline. In comparison, those requiring assistance with food intake showed substantially higher rates (22, 28, and 29%, respectively). Notably, patients fed via gastric tube or gastrostomy were at particularly high risk, with 56% experiencing PH. The differences were strongly statistically significant (*V* = 0.303, *p* < 0.001; *V* = 0.341, *p* < 0.001; *V* = 0.321, *p* = 0.002).

Physical activity was another strong predictor: only 4% of physically active patients had PH, 1% had 30-days complications, and 4% had 90-days functional decline. These rates were higher among those with limited activity (18, 6, and 10%) and those not physically active (17, 9, and 20%), with all differences being at least marginally significant (*V* = 0.200, *p* < 0.001; *V* = 0.165, *p* = 0.011; *V* = 0.204, *p* = 0.004).

Additional findings included a significantly increased risk of 30-days serious complications among patients with other than otorhinolaryngological oncological disorders (15.2% vs. 3.5%, *V* = 0.114, *p* = 0.002; see Supplementary Table S1). There was a trend toward more frequent PH among smokers and oncological patients, but these associations did not reach assumed significance level (*p* = 0.040 and 0.042, respectively). No significant differences were observed for gender or age.

Tables 4, 5 present characteristics and group comparisons of established risk and health status scales, including the Caprini score, Charlson Comorbidity Index (CCI), the ASA classification, VTE risk, Vulnerable Elders Survey-13 (VES-13), and a frailty scale (mFI-5). More detailed descriptive statistics are provided in Supplementary Table S2 and results of *U* tests are provided in Supplementary Table S3. Most patients in the study were classified as high risk on the Caprini and VTE scales, with 70% categorized as high risk for thrombosis (noting 17 missing VTE observations). The median Caprini score was 5 (mean ≈ 6), and a score of 5 or higher indicates a very high risk. Patients also had elevated CCI (median = 6, mean = 6.4) and ASA scores (median = 3, mean = 2.6), reflecting significant comorbidity and functional limitation. Notably, 80% of patients scored ≥ 3 on the VES-13, indicating high risk for loss of independence, though 157 observations were missing for this scale.

**Table 4:** Descriptive statistics of risk scales divided across groups of clinical outcomes and *χ*^2^ tests of independence for nominal risk variables.

Variable	Category	Total	Prolongedhospitalization		30-Daysserious complications		90-Daysfunctional decline	
			No	Yes	No	Yes	No	Yes
VES-13	< 3	54	50	4	38	1	32	1
		20.07%	92.59%	7.41%	97.44%	2.56%	96.97%	3.03%
	≥ 3	215	185	30	176	11	138	16
		79.93%	86.05%	13.95%	94.12%	5.88%	89.61%	10.39%
	Total	269	235	34	214	12	170	17
		100%	87.36%	12.64%	94.69%	5.31%	90.91%	9.09%
	*Χ*^2^(df), *p*-value		*Χ*^2^(1) = 1.675, *p* = 0.196		*Χ*^2^(1) = 0.707, *p* = 0.401		*Χ*^2^(1) = 1.781 *p* = 0.182	
	Cramer’s *V*		*V* = 0.079		*V* = 0.056		*V* = 0.098	
VTE	High risk (≥ 5 p)	299	258	41	244	15	181	24
		73.11%	86.29%	13.71%	94.21%	5.79%	88.29%	11.71%
	Moderate risk (3–4 p)	110	102	8	88	0	57	3
		26.89%	92.73%	7.27%	100.00%	0.00%	95.00%	5.00%
	Total	409	360	49	332	15	238	27
		100%	88.02%	11.98%	95.68%	4.32%	89.81%	10.19%
	*Χ*^2^(df), *p*-value		*Χ*^2^(1) = 3.162, *p* = 0.075		*Χ*^2^(1) = 5.327, *p* = 0.021		*Χ*^2^(1) = 2.282, *p* = 0.131	
	Cramer’s *V*		*V* = 0.088		*V* = 0.124		*V* = 0.093	
ASA (range: 1–5)	Missing	147	139	8	108	5	62	10
	Median	3	3	3	3	3	3	3
	Mean	2.638	2.622	2.732	2.593	3.000	2.556	3.000
	SD	0.570	0.574	0.549	0.549	0.447	0.540	0.343
Caprini (range: 3–18)	Missing	56	56	0	49	1	31	2
	Median	5	5	7	6	7	6	6
	Mean	5.849	5.698	6.837	5.875	7.400	6.046	7.038
	SD	2.119	2.072	2.183	2.067	2.293	2.159	3.218
CCI (range: 1–16)	Missing	113	105	8	88	5	65	4
	Median	6	6	7	6	9	6	7
	Mean	6.447	6.313	7.341	6.395	9.364	6.310	8.000
	SD	2.143	2.039	2.594	2.009	3.472	2.095	2.604
Frailty (range: 0–5)	Missing	182	169	13	152	6	83	8
	Median	2	2	2	2	2	2	2
	Mean	2.020	1.947	2.444	1.948	2.200	1.946	2.300
	SD	1.075	1.032	1.229	1.022	1.229	1.052	1.218

df, degrees of freedom; SD, standard deviation. Group of VTE moderate risk includes 1 patient with low risk (1–2 p) score. This group was merged with moderate risk group since one-observation group is too small to be a separate unit of analysis.

**Table 5:** Results of Welch *t*-tests of differences between groups of clinical outcomes in Caprini, CCI, ASA, and a frailty risk scales.

Comparison	Variable	*t*	df	*p*	Mean difference	Cohen’s *d*	99% CI for effect size	
							Lower	Upper
Prolonged hospitalization (Yes vs. No)	ASA	−1.176	56.132	0.245	−0.110	−0.196	−0.633	0.243
	Caprini	−3.425	61.936	0.001	−1.139	−0.535	−0.947	−0.119
	CCI	−2.429	47.733	0.019	−1.029	−0.441	−0.886	0.008
	Frailty	−2.292	43.947	0.027	−0.497	−0.438	−0.916	0.044
30-Days serious complications (Yes vs. No)	ASA	−2.916	11.454	0.014	−0.407	−0.812	−1.707	0.102
	Caprini	−2.525	15.180	0.023	−1.525	−0.699	−1.446	0.063
	CCI	−2.816	10.290	0.018	−2.969	−1.047	−2.026	−0.053
	Frailty	−0.637	9.659	0.539	−0.252	−0.223	−1.063	0.628
90-Days functional decline (Yes vs. No)	ASA	−4.934	25.931	< 0.001	−0.444	−0.982	−1.702	−0.251
	Caprini	−1.532	27.746	0.137	−0.993	−0.362	−0.908	0.190
	CCI	−3.053	27.023	0.005	−1.690	−0.715	−1.323	−0.099
	Frailty	−1.246	22.544	0.226	−0.354	−0.311	−0.929	0.313

df, degrees of freedom; CI, confidence interval.

When comparing patients with and without PH, those with longer stays had significantly higher Caprini scores (mean difference: 1.1 points; 6.8 vs. 5.7; *t*-test *p* = 0.001, Cohen’s *d* = 0.54; *U* test *p* < 0.001, rank biserial correlation = −0.34). The CCI was marginally significant (0.005 < *p* < 0.02; higher values in the PH group), while other scales were not significant. For 30-days serious complications, the ASA, Caprini, and CCI scores showed potentially significant differences (higher values in the serious complications group), with CCI demonstrating a strong effect size (Cohen’s *d* = −1.047, *t*-test *p* = 0.018) and having a significant result in *U* test (*p* = 0.002). For 90-days functional decline, both ASA and CCI were significant predictors (higher values in the functional decline group), with ASA showing a strong effect (*d* = −0.982, *p* < 0.001). Overall, univariate analyses suggest that CCI, Caprini, and ASA may help predict adverse clinical outcomes, with CCI appearing to be the most consistent predictor.

Logistic regression models were then constructed for each clinical outcome according to the procedure described in Materials and Methods Section. The coefficients of the models, as well as their quality measures are presented in Table 6. For PH, the best predictors were physical activity and independence in food intake. Physical activity significantly reduced the risk of PH compared to inactivity (OR = 0.222; [99% CI: 0.055–0.896]; *p* = 0.005), while food intake dependency increased the risk (OR = 2.974 [1.130–7.830], *p* = 0.004). The model showed moderate explanatory power (McFadden’s pseudo-*R*^2^ = 0.102; Nagelkerke’s pseudo-*R*^2^ = 0.139).

**Table 6:** Parameters and quality metrics of logistic regression models for three dependent variables, constructed using a full set of potential predictors (risk scales and functional independency variables).

Dependent variable	Independent variable	Estimate	Robust SE	OR	*z*	*p*	*R*^2^*_McF_* [*R*^2^*_N_*]	Δ*Χ*^2^ (df) *p_model_*	AIC vs. AIC_null_
Prolonged hospitalization	(Intercept)	−1.845	0.282	0.158	−6.532	< 0.001	0.102 [0.139]	29.42 (381) *p* < 0.001	268.21 vs. 291.63
	Physical activity: limited	0.044	0.347	1.045	0.126	0.899			
	Physical activity: yes	−1.507	0.542	0.222	−2.778	0.005			
	Dependent food intake	1.090	0.376	2.974	2.900	0.004			
30-Days serious complications	(Intercept)	−6.911	0.971	0.001	−7.121	< 0.001	0.321 [0.359]	29.39 (263) *p* < 0.001	68.23 vs. 93.62
	Dependent food intake	2.645	0.701	14.083	3.773	< 0.001			
	CCI	0.433	0.126	1.543	3.448	< 0.001			
90-Days functional decline	(Intercept)	−4.522	0.589	0.011	−7.684	< 0.001	0.199 [0.259]	29.59 (204) *p* < 0.001	127.19 vs. 150.77
	Independence: assisted living	0.230	0.594	1.409	0.388	0.698			
	Independence: dependent	3.408	0.892	30.203	3.821	< 0.001			
	CCI	0.343	0.086	1.259	3.982	< 0.001			

SE, standard error; OR, odds ratio; *R*^2^_McF_, McFadden’s pseudo-*R*^2^; *R*^2^_N_, Nagelkerke’s pseudo-*R*^2^; df, degrees of freedom; AIC, Akaike information criterion; AIC_null_, AIC of the model with no predictors (intercept only). Reference categories: physical activity—“no”; dependent food intake (consisting: with other person help, gastrostomy, and feeding tube)—“independently”; independence—“independent”. CCI was recoded by dividing 1 from all values to make 0 the minimum value, in order to make the intercepts interpretable.

For 30-days serious complications, the strongest predictors were dependence in food intake (OR = 14.083 [2.314–85.708], *p* < 0.001) and CCI (OR = 1.543 [1.116–2.132], *p* < 0.001), with high model fit (McFadden’s pseudo-*R*^2^ = 0.321; Nagelkerke’s pseudo-*R*^2^ = 0.359). The model for 90-days functional decline identified dependency as an extremely strong predictor (OR = 30.203 [3.036–300.425], *p* < 0.001), with CCI also significant (OR = 1.409 [1.129–1.759], *p* < 0.001; McFadden’s pseudo-*R*^2^ = 0.199; Nagelkerke’s pseudo-*R*^2^ = 0.259). Thus, all three models explained a substantial proportion of the variance in clinical outcomes.

To assess the predictive value of risk scales alone, additional models were constructed based on risk scales as only predictors included. The Caprini scale was the best single predictor risk scale of PH (*p* = 0.002), but its explanatory power was modest (McFadden’s pseudo-*R*^2^ = 0.036; Nagelkerke’s pseudo-*R*^2^ = 0.052). For 30-days serious complications, CCI alone was a strong predictor (*p* < 0.001; pseudo-*R*^2^ ≈ 0.17–0.19). For 90-days functional decline, both CCI and ASA were significant (*p* = 0.004 for both), and together explained a substantial proportion of the variance (pseudo-*R*^2^ ≈ 0.15–0.19). Notably, the Caprini and ASA scales were less effective predictors than patient characteristics, while CCI remained a robust predictor across 30-days serious complications and 90-days functional decline outcomes. Moreover, models based solely on patient functional status, that is, physical activity and dependency in daily functioning and food intake, outperformed models based on risk scales alone.

In summary, our analyses highlight the critical importance of functional status—operationalized as nutritional independence, independence in daily functioning and physical activity—as well as comorbidity burden, in predicting adverse clinical outcomes in the studied patient population. These findings underscore the need to incorporate comprehensive functional assessments into routine clinical evaluation and risk stratification.

## Discussion

This study explores the causes of hospital admissions among patients aged 80 years and older within the field of otorhinolaryngology, aiming to address the current gap in understanding the specific and evolving needs of this rapidly growing patient population. Older adults differ physiologically from younger individuals, and their disease profiles and therapeutic goals are distinct (6, 18). For geriatric patients, the preservation of functional abilities, autonomy, and quality of life are paramount, often superseding traditional curative aims (18). While the primary symptoms leading to the referral of geriatric patients from primary care to otorhinolaryngology are well characterized (19, 20), the detailed profile and complications of hospitalizations in those aged 80 and above remain underreported. Notably, much of the literature from the past decade still defines “elderly” as those aged 60 or 65, which no longer reflects the demographic reality of today’s aging societies (21). The pace of population aging has accelerated and the clinical challenges faced by the oldest old have evolved accordingly.

Emergencies and acute conditions constitute a substantial proportion of otolaryngology hospital admissions among older adults. However, direct comparisons across studies are hampered by differences in institutional protocols and the scarcity of research focusing exclusively on hospitalizations rather than outpatient or emergency department visits (22). Common indications for intervention in this population include facial trauma, epistaxis, foreign body impaction in the upper aerodigestive tract, infections or inflammatory processes (22, 23) and peripheral facial paralysis (24). Foreign body aspiration is particularly problematic in patients with dementia, where age-related impairments in swallowing and cough reflex further increase the risk and complexity of management (25–27). Inpatients blood transfusions are frequently required in older adults, reflecting their vulnerability to anemia and related conditions. In the material presented in the current study, emergencies account for 17% of all admissions, notably lower than those reported in the referenced literature. These emergency cases were distributed across ICD-10 groups R (acute inflammations, 6%), K (digestive system disorders, 5%), and T (injuries, 1%). Hemorrhage from the respiratory passages (R04) constituted 3% of cases, while difficulty breathing (R06) was observed in only 2% of the elderly cohort analyzed. Aging-related degenerative conditions such as hearing loss, tinnitus, or swallowing, accounted for less than 10% of cases, although other studies have demonstrated that these conditions contribute substantially to hospitalizations in this age group (23). The reason for the disproportion is the fact that in the Polish organizational system the treatment of these diseases is performed on an outpatient basis.

Cancer-related admissions requiring surgical intervention represent the vast majority of hospitalizations across eight institutions analyzed in the study. Notably, more than half of the patients in the analyzed cohort were assigned diagnostic codes corresponding to malignant neoplasms (ICD categories C and D). Direct comparisons of this proportional distribution with other patient populations remains challenging, primarily due to the limited availability of literature addressing such specific stratification. Nevertheless, it is important to highlight that major surgical procedures, including total laryngectomy and extensive ablative head and neck surgeries with microvascular reconstruction, are successfully performed within the age group of people under 80 (28–31). However, these interventions are frequently associated with a substantial burden of comorbidities, underscoring the need for meticulous patient selection and perioperative management to optimize clinical outcomes (25, 32–35).

Extended hospital stays are a well-documented phenomenon among elderly patients, primarily driven by increased susceptibility to complications and slower postoperative recovery. Previous studies have demonstrated that patients undergoing laryngectomy may require hospitalization ranging from 20 to 149 days (mean: 46 days), with higher CCI scores predicting significantly prolonged recovery (35). Postoperative complications are common in this population due to frailty and multiple comorbidities. In the previous study 51.3% of geriatric patients undergoing major head and neck procedures experienced serious complications within 30 days, and functional decline occurs in approximately 11% of previously independent individuals (30).

By integrating comprehensive patient-specific parameters and validated risk assessment scales, we identified key predictors of PH, thereby offering valuable insights for clinical risk stratification. Our cohort, obtained in eight different medical centers, rather representatively reflects the 80+ age group having otorhinolaryngological treatment, with 67% exposed to polypharmacy. The values of the Caprini, ASA, CCI scales were at the level of 5.8, 2.6, 6.4, reflecting the highrisk profile of the elderly group. Despite the advanced age and complex health profiles of these patients, the rates of PH, 30-days serious complications, and 90-days functional decline were relatively low (11.5, 4.5, and 10%, respectively).

The frequency of PH varied substantially, ranging from nearly 0 to 18% depending on the underlying reason for admission, however, this study does not prove statistical significance of these differences, hence, this issue requires further investigation on bigger and more heterogenous samples. We proved that the risk of PH, 30-days serious complications, and 90-days functional decline was markedly higher among those who were functionally dependent, required enteral feeding via nasogastric tube or gastrostomy or being no physically active. Importantly, three-quarters of the study sample were classified as high-risk for thrombosis, as indicated by elevated Caprini and VTE scores, and this risk was also positively correlated with PH These findings underscore a clinically significant feedback loop: patients at increased risk for thrombosis are more likely to experience extended hospital stays, which in turn may further elevate their thrombotic risk.

Lower rates of prolonged hospitalization and complications in our patient cohort, compared with other reports, may be explained by several complementary factors highlighted in recent literature. Many studies on elderly surgical populations report complication rates of 40–65%, particularly among frail or multimorbid patients undergoing major procedures (30, 36–39). In our study, rigorous functional assessment and patient selection, including systematic preoperative evaluation of nutritional status, physical activity, and independence, likely allowed for optimized and more selective surgical candidacy. Recent studies stress the impact of frailty and dependence on both complications and readmissions; lower rates in our cohort may reflect inclusion or intensified prehabilitation of frailer patients (40, 41). Furthermore, comprehensive multidisciplinary perioperative care, early mobilization, and nutritional support—key elements of modern perioperative protocols—are known to reduce complications and hospital stay in older adults (42). The experience of university and high-volume centers, where minimally invasive techniques are more common, may also have contributed to lower morbidity (43). Our cohort included a higher proportion of functionally independent, non-frail, or lower-CCI patients, and more elective than emergency procedures, which can reduce complication and hospitalization rates versus series dominated by more frail, urgent, or complex oncology cases (37, 44–47). Robust discharge planning and postoperative pathways enable achieving the higher rates of early discharge; structured follow-up may further drive down measured inpatient complications (48).

Finally, in our cohort PH was best predicted by lack of physical activity and food intake dependence. Risk scores such as Caprini and CCI had limited value for this outcome. 30-days serious complications were strongly associated with high CCI scores and food intake dependency. Combining comorbidity burden with functional status yielded the highest predictive accuracy. 90-days functional decline was most powerfully predicted by full dependency, with CCI offering additional prognostic value.

Our results highlight the necessity of incorporating functional status—particularly independence in mobility, food intake, and daily functioning—into routine risk assessment and management strategies in elderly hospitalized patients, as traditional scales alone may fail to reliably estimate the risk of PH and other adverse outcomes. Models based solely on risk scales remained markedly inferior to those incorporating functional variables.

Of the evaluated tools, only CCI scale consistently added relevant prognostic value, particularly for 30-days serious complications and 90-days functional decline. This finding highlights the importance of comorbidity burden in shaping outcomes among elderly otolaryngology inpatients, where multimorbidity and frailty frequently coexist. The CCI likely captures the cumulative effect of chronic conditions such as cardiovascular disease, diabetes, and pulmonary disorders, which increase susceptibility to perioperative complications and limit recovery potential. Nonetheless, the explanatory power of CCI alone was limited compared with models that integrated functional parameters such as physical activity, food intake, and independence in daily living. Thus, while CCI remains a useful adjunct in geriatric risk assessment, its greatest utility in this population may lie in combination with functional measures.

The principal strength of our study is its multidimensional approach to identify how clinical, demographic, and functional characteristics are associated with three adverse events: PH, 30-days serious complications and 90-days functional decline within our geriatric cohort. Additionally, the study examines both variable-to-variable and multivariate associations to determine which factors are most strongly linked to these outcomes A notable strength of our study is the relatively large cohort of patients aged 80 and above, made possible by the multicenter design. This approach enabled us to perform first comprehensive multivariate analyses of hospitalization profiles among patients aged 80 and above in otolaryngology. Crucially, the study correlates baseline patient status with in-hospital complications through multivariate modeling, allowing for an in-depth evaluation of clinical, demographic, and perioperative risk factors. As the demographic landscape continues to shift, the urgent need for contemporary research and individualized clinical prehabilitation pathways becomes increasingly apparent, with the aim of optimizing outcomes and mitigating the phenomenon of PH in this particularly vulnerable cohort (41, 49, 50).

Prehabilitation, including interventions aimed at improving functional status prior to surgery, plays an increasingly significant role in optimizing clinical outcomes among elderly patients, notably those over the age of 80 (51). A review of contemporary literature demonstrates that implementation of prehabilitation programs (41), including physical exercise, nutritional support, and psychoeducational interventions, can effectively reduce the overall incidence of postoperative complications, enhance functional reserves, and accelerate the post-surgical recovery process in elderly patients, while significantly improving quality of life (52). Prehabilitation should be considered an integral component of preparing elderly patients for surgical intervention, particularly those with decreased functional reserve. Practical, evidence-based recommendations remain limited due to the relatively small number of studies directly focusing on octogenarians and insufficient data regarding the impact of prehabilitation on the most severe complications and survival rates (53). Prehabilitation programs should comprise comprehensive patient assessment, individualized interventions, and multidisciplinary collaboration involving physiotherapists, dietitians, and psychologists (54, 55).

Despite the strengths of multicenter design employed, our study has several limitations. The most direct limitation relates to numerous missing data. A multicenter design causes some uncontrolled variability in how data were reported across participating centers. This variability likely affected data quality and contributed to missing values. While these gaps were transparently documented at each stage of analysis—through number of valid and missing values and degrees of freedom—they limit the strength of some conclusions as it can render estimators of some effects biased. Furthermore, nearly 150 missing observations in the 90-days follow-up data may have led to underestimation of functional decline and the bias of the relationship between functional decline and other variables. To explore this issue further, we assessed additional clinical and administrative data to better characterize the missing cases. Specifically, we examined ICD-10 diagnostic codes with attention to their clinical significance for long-term outcomes (e.g., patients treated for a foreign body in the larynx, who after removal typically do not experience lasting complications). We also reviewed CCI scores, surgical complication reports, indicators of PH, emergency admission status, results of the G8 questionnaire, and subjective general health ratings. These data sources allowed us to estimate the likelihood of 90-days functional decline where direct follow-up was unavailable. Based on this multi-layered assessment, cases were categorized as probably not associated with functional decline, requiring further consideration, or likely to represent functional decline.

This additional analysis suggested that of the 149 data gaps, approximately 40–45% could be classified as probably not associated with functional decline, approximately 15% as probable (though not certain) functional decline, and the remainder as impossible to determine. Assuming that most of the probable declines were indeed declined and that the cases that could not be determined had a similar distribution to the valid data (approximately 10% of functional declines), this means that there would be approximately 10–20% functional declines among patients for whom data were missing. Thus the actual scale of 90-days functional decline may be several percentage points higher than reported in the results. However, the possible difference between the reported distribution of this variable and the roughly estimated distribution among the missing data is not particularly large. This additional analysis suggests that most likely, missing data on functional decline did not follow a specific, completely different pattern, which is in favor of the presented results. However, it is advisable to treat the part of the results related to this variable with caution.

Other limitations of the study should also be considered. The exploratory nature of our analysis and many comparisons tested increase the risk of Type I error; although this was mitigated by using a stricter significance level, it should still be acknowledged—fortunately, the key associations (physical activity, dependency, and food intake) were consistently observed across all outcomes, making it unlikely that this limitation undermines the main conclusions. Another limitation is the lack of standardized assessment tools across centers when recording functional status (e.g., independence or activity level), which may have introduced variability in how these variables were interpreted or documented.

The findings of this study carry important implications for routine clinical decision-making. In otolaryngology and geriatric care settings, prioritizing assessments of physical activity, food intake independence, and general functional status may allow earlier identification of high-risk patients—particularly those at risk of complications or PH. Embedding simple functional screens into admission protocols could inform treatment planning, help avoid unnecessary interventions, and guide perioperative optimization, especially in surgical oncology cases. Given that traditional tools such as ASA and Caprini scores were less informative, functional metrics may better support tailored care pathways for the “oldest old” otolaryngology inpatients.

## Data Availability

The raw data supporting the conclusions of this article will be made available by the authors, without undue reservation.

## Footnotes

^1^ https://www.who.int/news-room/fact-sheets/detail/ageing-and-health
