# Supplementary material for: Functional dependence predicts adverse outcomes among geriatric otolaryngology patients better than more complex risk scales: a multivariate analysis of hospitalization risks on elderly group
Source: Front Med (Lausanne). 2025 Dec 11;12:1690442. doi: 10.3389/fmed.2025.1690442 (PMC12771765; doi:10.3389/fmed.2025.1690442)
Supplement: Supplementary file 1 [file Data_Sheet_1.docx]

**Table S1. Descriptive statistics and results of *χ²* tests of independence of all variables included in the study across groups of clinical outcomes.**

| **Variable** | **Category** | **Total** | **Prolonged hospitalization** | | **30-Days**  **serious complications** | | **90-Days**  **functional decline** | |
| --- | --- | --- | --- | --- | --- | --- | --- | --- |
|  |  |  | no | yes | no | yes | no | yes |
| **Gender** | female | 213 | 189 | 24 | 168 | 7 | 125 | 10 |
|  |  | 50.00% | 88.73% | 11.27% | 96.00% | 4.00% | 92.59% | 7.41% |
|  | male | 213 | 188 | 25 | 176 | 9 | 124 | 18 |
|  |  | 50.00% | 88.26% | 11.74% | 95.14% | 4.87% | 87.32% | 12.68% |
|  | Total | 426 | 377 | 49 | 344 | 16 | 249 | 28 |
|  |  | 100.00% | 88.50% | 11.50% | 95.56% | 4.44% | 89.89% | 10.11% |
|  | *Χ²*(df), *p*-value | | *Χ²*(1) = 0.023, *p* = 0.879 | | *Χ²*(1) = 0.158, *p* = 0.691 | | *Χ²*(1) = 2.114, *p* = 0.146 | |
|  | Cramer’s *V* | | *V* = 0.007 | | *V* = 0.021 | | *V* = 0.087 | |
| **Independence** | independent | 307 | 279 | 28 | 265 | 6 | 192 | 13 |
|  |  | 72.58% | 90.88% | 9.12% | 97.79% | 2.21% | 93.66% | 6.34% |
|  | assisted living | 91 | 77 | 14 | 65 | 7 | 52 | 8 |
|  |  | 21.51% | 84.62% | 15.39% | 90.28% | 9.72% | 86.67% | 13.33% |
|  | dependent | 25 | 18 | 7 | 12 | 3 | 3 | 7 |
|  |  | 5.91% | 72.00% | 28.00% | 80.00% | 20.00% | 30.00% | 70.00% |
|  | Total | 423 | 374 | 49 | 342 | 16 | 247 | 28 |
|  |  | 100.00% | 88.42% | 11.58% | 95.53% | 4.47% | 89.82% | 10.18% |
|  | *Χ²*(df), *p*-value | | *Χ²*(2) = 9.68, *p* = 0.008 | | *Χ²*(2) = 16.356, *p* < 0.001 | | *Χ²*(2) = 43.085, *p* < 0.001 | |
|  | Cramer’s *V* | | *V* = 0.151 | | *V* = 0.214 | | *V* = 0.396 | |
| **Food intake** | independently | 364 | 333 | 31 | 315 | 8 | 232 | 21 |
|  |  | 87.29% | 91.48% | 8.52% | 97.52% | 2.48% | 91.70% | 8.30% |
|  | with other person help | 37 | 29 | 8 | 18 | 7 | 10 | 4 |
|  |  | 8.87% | 78.38% | 21.62% | 72.00% | 28.00% | 71.43% | 28.57% |
|  | gastrostomy | 9 | 4 | 5 | 8 | 0 | 4 | 3 |
|  |  | 2.16% | 44.44% | 55.56% | 100.00% | 0.00% | 57.14% | 42.86% |
|  | feeding tube | 7 | 3 | 4 | 2 | 1 | 3 | 0 |
|  |  | 1.68% | 42.86% | 57.14% | 66.67% | 33.33% | 100.00% | 0.00% |
|  | Total | 417 | 369 | 48 | 343 | 16 | 249 | 28 |
|  |  | 100.00% | 88.49% | 11.51% | 95.54% | 4.46% | 89.89% | 10.11% |
|  | *Χ²*(df), *p*-value | | *Χ²*(3) = 38.369, *p* < 0.001 | | *Χ²*(3) = 41.764, *p* < 0.001 | | *Χ²*(3) = 14.762, *p* = 0.002 | |
|  | Cramer’s *V* | | *V* = 0.303 | | *V* = 0.341 | | *V* = 0.231 | |
| **Polypharmacy** | 0 | 26 | 22 | 4 | 19 | 1 | 13 | 2 |
|  |  | 6.57% | 84.62% | 15.39% | 95.00% | 5.00% | 86.67% | 13.33% |
|  | ≤ 3 | 103 | 93 | 10 | 93 | 2 | 66 | 3 |
|  |  | 26.01% | 90.29% | 9.71% | 97.90% | 2.11% | 95.65% | 4.35% |
|  | > 3 | 267 | 236 | 31 | 214 | 11 | 160 | 20 |
|  |  | 67.42% | 88.39% | 11.61% | 95.11% | 4.89% | 88.89% | 11.11% |
|  | Total | 396 | 351 | 45 | 326 | 14 | 239 | 25 |
|  |  | 100.00% | 88.64% | 11.36% | 95.88% | 4.12% | 90.53% | 9.47% |
|  | *Χ²*(df), *p*-value | | *Χ²*(2) = 0.714, *p* = 0.7 | | *Χ²*(2) = 1.353, *p* = 0.508 | | *Χ²*(2) = 2.938, *p* = 0.23 | |
|  | Cramer’s *V* | | *V* = 0.042 | | *V* = 0.063 | | *V* = 0.105 | |
| **Physical activity** | yes | 147 | 141 | 6 | 131 | 1 | 92 | 4 |
|  |  | 37.79% | 95.92% | 4.08% | 99.24% | 0.76% | 95.83% | 4.17% |
|  | limited | 138 | 113 | 25 | 104 | 7 | 82 | 9 |
|  |  | 35.48% | 81.88% | 18.12% | 93.69% | 6.31% | 90.11% | 9.89% |
|  | no | 104 | 86 | 18 | 78 | 8 | 61 | 15 |
|  |  | 26.74% | 82.69% | 17.31% | 90.70% | 9.30% | 80.26% | 19.74% |
|  | Total | 389 | 340 | 49 | 313 | 16 | 235 | 28 |
|  |  | 100.00% | 87.40% | 12.60% | 95.14% | 4.86% | 89.35% | 10.65% |
|  | *Χ²*(df), *p*-value | | *Χ²*(2) = 15.596, *p* < 0.001 | | *Χ²*(2) = 8.972, *p* = 0.011 | | *Χ²*(2) = 10.894, *p* = 0.004 | |
|  | Cramer’s *V* | | *V* = 0.2 | | *V* = 0.165 | | *V* = 0.204 | |

**Table S1. (Continued)**

| **Variable** | **Category** | **Total** | **Prolonged hospitalization** | | **30-Days**  **serious complications** | | **90-Days**  **functional decline** | |
| --- | --- | --- | --- | --- | --- | --- | --- | --- |
|  |  |  | no | yes | no | yes | no | yes |
| **Alcohol** | no | 305 | 270 | 35 | 249 | 12 | 195 | 18 |
|  |  | 93.85% | 88.53% | 11.48% | 95.40% | 4.60% | 91.55% | 8.45% |
|  | yes | 20 | 17 | 3 | 17 | 2 | 9 | 2 |
|  |  | 6.15% | 85.00% | 15.00% | 89.47% | 10.53% | 81.82% | 18.18% |
|  | Total | 325 | 287 | 38 | 266 | 14 | 204 | 20 |
|  |  | 100.00% | 88.31% | 11.69% | 95.00% | 5.00% | 91.07% | 8.93% |
|  | *Χ²*(df), *p*-value | | *Χ²*(1) = 0.226, *p* = 0.635 | | *Χ²*(1) = 1.311, *p* = 0.252 | | *Χ²*(1) = 1.218, *p* = 0.270 | |
|  | Cramer’s *V* | | *V* = 0.026 | | *V* = 0.068 | | *V* = 0.074 | |
| **Smoking** | no | 225 | 206 | 19 | 179 | 7 | 129 | 12 |
|  |  | 59.37% | 91.56% | 8.44% | 96.24% | 3.76% | 91.49% | 8.51% |
|  | yes in past | 123 | 103 | 20 | 107 | 7 | 90 | 10 |
|  |  | 32.45% | 83.74% | 16.26% | 93.86% | 6.14% | 90.00% | 10.00% |
|  | yes currently | 31 | 25 | 6 | 27 | 0 | 15 | 2 |
|  |  | 8.18% | 80.65% | 19.36% | 100.00% | 0.00% | 88.24% | 11.77% |
|  | Total | 379 | 334 | 45 | 313 | 14 | 234 | 24 |
|  |  | 100.00% | 88.13% | 11.87% | 95.72% | 4.28% | 90.70% | 9.30% |
|  | *Χ²*(df), *p*-value | | *Χ²*(2) = 6.449, *p* = 0.040 | | *Χ²*(2) = 2.291, *p* = 0.318 | | *Χ²*(2) = 0.285, *p* = 0.867 | |
|  | Cramer’s *V* | | *V* = 0.13 | | *V* = 0.084 | | *V* = 0.033 | |
| **Oncological patient** | no | 219 | 200 | 19 | 174 | 5 | 120 | 10 |
|  |  | 52.39% | 91.32% | 8.68% | 97.21% | 2.79% | 92.31% | 7.69% |
|  | yes | 199 | 169 | 30 | 167 | 10 | 126 | 17 |
|  |  | 47.61% | 84.93% | 15.08% | 94.35% | 5.65% | 88.11% | 11.89% |
|  | Total | 418 | 369 | 49 | 341 | 15 | 246 | 27 |
|  |  | 100.00% | 88.28% | 11.72% | 95.79% | 4.21% | 90.11% | 9.89% |
|  | *Χ²*(df), *p*-value | | *Χ²*(1) = 4.126, *p* = 0.042 | | *Χ²*(1) = 1.799, *p* = 0.180 | | *Χ²*(1) = 1.345, *p* = 0.246 | |
|  | Cramer’s *V* | | *V* = 0.099 | | *V* = 0.071 | | *V* = 0.07 | |
| **Oncological disorders currently (other than ORL)** | no | 375 | 331 | 44 | 308 | 11 | 222 | 24 |
|  |  | 90.80% | 88.27% | 11.73% | 96.55% | 3.45% | 90.24% | 9.76% |
|  | yes | 38 | 34 | 4 | 28 | 5 | 23 | 4 |
|  |  | 9.20% | 89.47% | 10.53% | 84.85% | 15.15% | 85.19% | 14.82% |
|  | Total | 413 | 365 | 48 | 336 | 16 | 245 | 28 |
|  |  | 100.00% | 88.38% | 11.62% | 95.46% | 4.55% | 89.74% | 10.26% |
|  | *Χ²*(df), *p*-value | | *Χ²*(1) = 0.049, *p* = 0.825 | | *Χ²*(1) = 9.441, *p* = 0.002 | | *Χ²*(1) = 0.676, *p* = 0.411 | |
|  | Cramer’s *V* | | *V* = 0.011 | | *V* = 0.164 | | *V* = 0.05 | |
| **Oncological family history** | no | 146 | 131 | 15 | 131 | 4 | 96 | 9 |
|  |  | 76.84% | 89.73% | 10.27% | 97.04% | 2.96% | 91.43% | 8.57% |
|  | yes | 44 | 42 | 2 | 40 | 2 | 24 | 2 |
|  |  | 23.16% | 95.46% | 4.55% | 95.24% | 4.76% | 92.31% | 7.69% |
|  | Total | 190 | 173 | 17 | 171 | 6 | 120 | 11 |
|  |  | 100.00% | 91.05% | 8.95% | 96.61% | 3.39% | 91.60% | 8.40% |
|  | *Χ²*(df), *p*-value | | *Χ²*(1) = 1.362, *p* = 0.243 | | *Χ²*(1) = 0.317, *p* = 0.574 | | *Χ²*(1) = 0.021, *p* = 0.885 | |
|  | Cramer’s *V* | | *V* = 0.085 | | *V* = 0.042 | | *V* = 0.013 | |
| **Oncological disorders in past** | no | 267 | 238 | 29 | 224 | 8 | 172 | 16 |
|  |  | 71.01% | 89.14% | 10.86% | 96.55% | 3.45% | 91.49% | 8.51% |
|  | yes | 109 | 97 | 12 | 94 | 3 | 60 | 6 |
|  |  | 28.99% | 88.99% | 11.01% | 96.91% | 3.09% | 90.91% | 9.09% |
|  | Total | 376 | 335 | 41 | 318 | 11 | 232 | 22 |
|  |  | 100.00% | 89.10% | 10.90% | 96.66% | 3.34% | 91.34% | 8.66% |
|  | *Χ²*(df), *p*-value | | *Χ²*(1) = 0.002, *p* = 0.967 | | *Χ²*(1) = 0.027, *p* = 0.870 | | *Χ²*(1) = 0.021, *p* = 0.885 | |
|  | Cramer’s *V* | | *V* = 0.002 | | *V* = 0.009 | | *V* = 0.009 | |

**Table S2.** **Detailed descriptive statistics of risk scales divided across** **groups of clinical outcomes and *χ²* tests of independence for nominal risk variables.**

| **Variable** | **Category** | **Total** | **Prolonged**  **hospitalization** | | **30-Days**  **serious complications** | | **90-Days**  **functional decline** | |
| --- | --- | --- | --- | --- | --- | --- | --- | --- |
|  |  |  | no | yes | no | yes | no | yes |
| **VES-13** | < 3 | 54 | 50 | 4 | 38 | 1 | 32 | 1 |
|  |  | 20.07% | 92.59% | 7.41% | 97.44% | 2.56% | 96.97% | 3.03% |
|  | ≥ 3 | 215 | 185 | 30 | 176 | 11 | 138 | 16 |
|  |  | 79.93% | 86.05% | 13.95% | 94.12% | 5.88% | 89.61% | 10.39% |
|  | Total | 269 | 235 | 34 | 214 | 12 | 170 | 17 |
|  |  | 100% | 87.36% | 12.64% | 94.69% | 5.31% | 90.91% | 9.09% |
|  | *Χ²*(df), *p*-value | | *Χ²*(1) = 1.675, *p* = 0.196 | | *Χ²*(1) = 0.707, *p* = 0.401 | | *Χ²*(1) = 1.781 *p* = 0.182 | |
|  | Cramer’s *V* | | *V* = 0.079 | | *V* = 0.056 | | *V* = 0.098 | |
| **VTE** | high risk  (≥ 5p) | 299 | 258 | 41 | 244 | 15 | 181 | 24 |
|  |  | 73.11% | 86.29% | 13.71% | 94.21% | 5.79% | 88.29% | 11.71% |
|  | moderate risk (3–4p) | 110 | 102 | 8 | 88 | 0 | 57 | 3 |
|  |  | 26.89% | 92.73% | 7.27% | 100.00% | 0.00% | 95.00% | 5.00% |
|  | Total | 409 | 360 | 49 | 332 | 15 | 238 | 27 |
|  |  | 100% | 88.02% | 11.98% | 95.68% | 4.32% | 89.81% | 10.19% |
|  | *Χ²*(df), *p*-value | | *Χ²*(1) = 3.162, *p* = 0.075 | | *Χ²*(1) = 5.327, *p* = 0.021 | | *Χ²*(1) = 2.282, *p* = 0.131 | |
|  | Cramer’s *V* | | *V* = 0.088 | | *V* = 0.124 | | *V* = 0.093 | |
| **ASA** | Valid | 279 | 238 | 41 | 236 | 11 | 187 | 18 |
|  | Missing | 147 | 139 | 8 | 108 | 5 | 62 | 10 |
|  | Minimum | 1 | 1 | 2 | 1 | 2 | 1 | 2 |
|  | Maximum | 5 | 5 | 4 | 5 | 4 | 4 | 4 |
|  | 1st quartile | 2 | 2 | 2 | 2 | 3 | 2 | 3 |
|  | Median | 3 | 3 | 3 | 3 | 3 | 3 | 3 |
|  | 3rd quartile | 3 | 3 | 3 | 3 | 3 | 3 | 3 |
|  | Mean | 2.638 | 2.622 | 2.732 | 2.593 | 3.000 | 2.556 | 3.000 |
|  | SD | 0.57 | 0.574 | 0.549 | 0.549 | 0.447 | 0.540 | 0.343 |
|  | Skewness | 0.204 | 0.255 | -0.081 | 0.012 | 0 | -0.226 | 0 |
| **Caprini** | Valid | 370 | 321 | 49 | 295 | 15 | 218 | 26 |
|  | Missing | 56 | 56 | 0 | 49 | 1 | 31 | 2 |
|  | Minimum | 3 | 3 | 3 | 3 | 5 | 3 | 3 |
|  | Maximum | 18 | 18 | 13 | 17 | 13 | 17 | 18 |
|  | 1st quartile | 4 | 4 | 5 | 4 | 6 | 5 | 5 |
|  | Median | 5 | 5 | 7 | 6 | 7 | 6 | 6 |
|  | 3rd quartile | 7 | 7 | 8 | 7 | 8.5 | 7 | 7.750 |
|  | Mean | 5.849 | 5.698 | 6.837 | 5.875 | 7.400 | 6.046 | 7.038 |
|  | SD | 2.119 | 2.072 | 2.183 | 2.067 | 2.293 | 2.159 | 3.218 |
|  | Skewness | 1.697 | 1.941 | 0.706 | 1.418 | 1.113 | 1.354 | 1.975 |
| **CCI** | Valid | 313 | 272 | 41 | 256 | 11 | 184 | 24 |
|  | Missing | 113 | 105 | 8 | 88 | 5 | 65 | 4 |
|  | Minimum | 1 | 1 | 4 | 1 | 6 | 1 | 5 |
|  | Maximum | 16 | 14 | 16 | 13 | 16 | 13 | 16 |
|  | 1st quartile | 5 | 5 | 6 | 5 | 6 | 5 | 6 |
|  | Median | 6 | 6 | 7 | 6 | 9 | 6 | 7 |
|  | 3rd quartile | 8 | 8 | 8 | 8 | 11 | 8 | 10 |
|  | Mean | 6.447 | 6.313 | 7.341 | 6.395 | 9.364 | 6.310 | 8.000 |
|  | SD | 2.143 | 2.039 | 2.594 | 2.009 | 3.472 | 2.095 | 2.604 |
|  | Skewness | 1.008 | 0.863 | 1.229 | 0.776 | 0.747 | 0.855 | 1.402 |

**Table S2. (Continued)**

| **Variable** | **Category** | **Total** | **Prolonged**  **hospitalization** | | **30-Days**  **serious complications** | | **90-Days**  **functional decline** | |
| --- | --- | --- | --- | --- | --- | --- | --- | --- |
|  |  |  | no | yes | no | yes | no | yes |
| **Frailty** | Valid | 244 | 208 | 36 | 192 | 10 | 166 | 20 |
|  | Missing | 182 | 169 | 13 | 152 | 6 | 83 | 8 |
|  | Minimum | 0 | 0 | 1 | 0 | 1 | 0 | 1 |
|  | Maximum | 5 | 5 | 5 | 5 | 4 | 5 | 4 |
|  | 1st quartile | 1 | 1 | 1.750 | 1 | 1 | 1 | 1 |
|  | Median | 2 | 2 | 2 | 2 | 2 | 2 | 2 |
|  | 3rd quartile | 3 | 3 | 3 | 3 | 3 | 3 | 3.250 |
|  | Mean | 2.020 | 1.947 | 2.444 | 1.948 | 2.200 | 1.946 | 2.300 |
|  | SD | 1.075 | 1.032 | 1.229 | 1.022 | 1.229 | 1.052 | 1.218 |
|  | Skewness | 0.640 | 0.586 | 0.624 | 0.670 | 0.431 | 0.679 | 0.326 |

***Note*. Group of VTE moderate risk includes 1 patient with low risk (1–2p) score. This group was merged with moderate risk group since one-observation group is too small to be a separate unit of analysis.**

**Table S3.** **Welch *t*-tests and Mann-Whitney-Wilcoxon *U* tests of differences between groups of clinical outcomes in** **Caprini, CCI, ASA, and a frailty risk scales.**

|  |  | | | | | | | **99% CI for effect size** | |
| --- | --- | --- | --- | --- | --- | --- | --- | --- | --- |
| **Comparison** | **Variable** | **Test** | **Statistic** | **df** | ***p*** | **Location parameter** | **Effect size** | **Lower** | **Upper** |
| **Prolonged hospitalization** (yes vs no) | ASA | Welch | -1.176 | 56.132 | 0.245 | -0.110 | -0.196 | -0.633 | 0.243 |
|  |  | MW | 4394.5 |  | 0.239 | 0 | -0.099 | -0.338 | 0.151 |
|  | Caprini | Welch | -3.425 | 61.936 | 0.001 | -1.139 | -0.535 | -0.947 | -0.119 |
|  |  | MW | 5191.5 |  | < 0.001 | -1 | -0.340 | -0.524 | -0.125 |
|  | CCI | Welch | -2.429 | 47.733 | 0.019 | -1.029 | -0.441 | -0.886 | 0.008 |
|  |  | MW | 4251.5 |  | 0.013 | -1 | -0.238 | -0.456 | 0.007 |
|  | Frailty | Welch | -2.292 | 43.947 | 0.027 | -0.497 | -0.438 | -0.916 | 0.044 |
|  |  | MW | 2914 |  | 0.026 | 0 | -0.222 | -0.458 | 0.044 |
| **30-Days serious complications** (yes vs no) | ASA | Welch | -2.916 | 11.454 | 0.014 | -0.407 | -0.812 | -1.707 | 0.102 |
|  |  | MW | 822.5 |  | 0.017 | -50.03 | -0.366 | -0.688 | 0.075 |
|  | Caprini | Welch | -2.525 | 15.180 | 0.023 | -1.525 | -0.699 | -1.446 | 0.063 |
|  |  | MW | 1295.5 |  | 0.006 | -1 | -0.414 | -0.683 | -0.047 |
|  | CCI | Welch | -2.816 | 10.290 | 0.018 | -2.969 | -1.047 | -2.026 | -0.053 |
|  |  | MW | 641.5 |  | 0.002 | -2 | -0.544 | -0.789 | -0.150 |
|  | Frailty | Welch | -0.637 | 9.659 | 0.539 | -0.252 | -0.223 | -1.063 | 0.628 |
|  |  | MW | 860 |  | 0.562 | -15.26 | -0.104 | -0.529 | 0.362 |
| **90-Days functional decline**  (yes vs no) | ASA | Welch | -4.934 | 25.931 | < 0.001 | -0.444 | -0.982 | -1.702 | -0.251 |
|  |  | MW | 993.5 |  | < 0.001 | -17.45 | -0.410 | -0.666 | -0.067 |
|  | Caprini | Welch | -1.532 | 27.746 | 0.137 | -0.993 | -0.362 | -0.908 | 0.190 |
|  |  | MW | 2347.5 |  | 0.147 | -1 | -0.172 | -0.448 | 0.135 |
|  | CCI | Welch | -3.053 | 27.023 | 0.005 | -1.69 | -0.715 | -1.323 | -0.099 |
|  |  | MW | 1291 |  | < 0.001 | -2 | -0.415 | -0.644 | -0.118 |
|  | Frailty | Welch | -1.246 | 22.544 | 0.226 | -0.354 | -0.311 | -0.929 | 0.313 |
|  |  | MW | 1400.5 |  | 0.232 | -49.39 | -0.156 | -0.470 | 0.193 |

***Note*. Effect size is defined as Cohen’s *d* for Welch *t*-test and rank-biserial correlation for *U* test.**
